# Zymogenic latency in an ∼250-million-year-old astacin metallopeptidase

**DOI:** 10.1107/S2059798322009688

**Published:** 2022-10-20

**Authors:** Tibisay Guevara, Arturo Rodríguez-Banqueri, Walter Stöcker, Christoph Becker-Pauly, F. Xavier Gomis-Rüth

**Affiliations:** a Proteolysis Laboratory, Department of Structural Biology, Molecular Biology Institute of Barcelona (IBMB), Higher Scientific Research Council (CSIC), Barcelona Science Park, Baldiri Reixac 15–21, Helix Building, 08028 Barcelona, Catalonia, Spain; b Institut für Molekulare Physiologie (IMP), Johannes-Gutenberg Universität Mainz (JGU), Johann-Joachim-Becher-Weg 7, 55128 Mainz, Germany; c Biochemical Institute, Christian-Albrechts-Universität zu Kiel, Otto-Hahn-Platz 9, 24118 Kiel, Germany; Lund University, Sweden

**Keywords:** metallopeptidase zymogenic latency, astacin metallopeptidase, *Limulus polyphemus*, horseshoe crab, aspartate-switch mechanism, catalytic domain, pro-peptide

## Abstract

The horseshoe crab *Limulus polyphemus* is one of few extant *Limulus* species, which date back to ∼250 million years ago under the conservation of a common *Bauplan* documented by fossil records. It possesses the only proteolytic blood-coagulation and innate immunity system outside vertebrates and is a model organism for the study of the evolution and function of peptidases. The astacins are a family of metallopeptidases that share a central ∼200-residue catalytic domain (CD), which is found in >1000 species across holozoans and, sporadically, bacteria. Here, the zymogen of an astacin from *L. polyphemus* was crystallized and its structure was solved. A 34-residue, mostly unstructured pro-peptide (PP) traverses, and thus blocks, the active-site cleft of the CD in the opposite direction to a substrate. A central ‘PP motif’ (F^35^-E-G-D-I^39^) adopts a loop structure which positions Asp38 to bind the catalytic metal, replacing the solvent molecule required for catalysis in the mature enzyme according to an ‘aspartate-switch’ mechanism. Maturation cleavage of the PP liberates the cleft and causes the rearrangement of an ‘activation segment’. Moreover, the mature N-terminus is repositioned to penetrate the CD moiety and is anchored to a buried ‘family-specific’ glutamate. Overall, this mechanism of latency is reminiscent of that of the other three astacins with known zymogenic and mature structures, namely crayfish astacin, human meprin β and bacterial myroilysin, but each shows specific structural characteristics. Remarkably, myroilysin lacks the PP motif and employs a cysteine instead of the aspartate to block the catalytic metal.

## Abstract 2

The horseshoe crab *Limulus polyphemus* is an ancient chelicerate that is a model organism for the study of the evolution and function of peptidases. It contains a member of the astacin metallopeptidases, the mechanism of latency of which was revealed by X-ray structural analysis.

## 1. Introduction

The Atlantic horseshoe crab *Limulus polyphemus* (Linnaeus, 1758 ▸) is a unique marine merostomatous decapod that is endemic to North America (Shuster, 1982 ▸; Walls *et al.*, 2002 ▸). It is one of four closely related extant species of horseshoe crabs together with *Tachypleus tridentatus*, *Tachypleus gigas* and *Carcinoscorpius rotundicaudia*, which are found in Asia (Sekiguchi & Shuster, 2009 ▸). They are the only survivors of the order Xiphosurida (Bicknell & Pates, 2020 ▸) and are the closest living relatives of trilobites (Shuster, 1982 ▸). Indeed, *Limulus* spp. go back to ∼250 million years ago (Mya) (Bicknell & Pates, 2020 ▸) and the Limulidae family has existed since the Carboniferous period (∼360 Mya; Bicknell & Pates, 2019 ▸, 2020 ▸). Xiphosurida, which share a highly conserved horseshoe-crab-like *Bauplan* as inferred from an exceptionally extensive fossil record (Bicknell & Pates, 2020 ▸), date as far back as the Late Ordovician (∼445 Mya; Rudkin *et al.*, 2008 ▸; Bicknell & Pates, 2020 ▸) or Cambrian (∼540 Mya; Størmer, 1952 ▸). Thus, these animals have survived all five great mass extinctions and are sometimes considered to be ‘living fossils’, a term introduced by Charles Darwin (Darwin, 1859 ▸), or ‘stabilomorphs’ (Kin & Błażejowski, 2014 ▸) and are an example of ‘evolutionary stasis’ (Rudkin *et al.*, 2008 ▸).

Despite their name, horseshoe crabs are actually not crustaceans but chelicerates that are phylogenetically closer to spiders, ticks and scorpions than to crabs (Lankester, 1881 ▸; Ballesteros & Sharma, 2019 ▸). *L. polyphemus* has a remarkable estimated life expectancy of up to 20 years (Walls *et al.*, 2002 ▸) and is frequently used as a laboratory animal model to study its compound eyes, its simple nervous system and marine invertebrate embryology in general (Smith, 2022 ▸). Moreover, it possesses an ancient and primitive proteolytic blood-coagulation and innate immunity system, which is the only one found outside vertebrates (Rowley *et al.*, 1984 ▸; Doolittle, 2010 ▸; Schmid *et al.*, 2019 ▸; Winter *et al.*, 2020 ▸; Eleftherianos *et al.*, 2021 ▸). Thus, *L. polyphemus* is an important organism for study of the evolution and function of peptidases (Becker-Pauly *et al.*, 2009 ▸).

The astacins are a family of zinc-dependent metallopeptidases (MPs; Stöcker *et al.*, 1993 ▸; Gomis-Rüth, Trillo-Muyo *et al.*, 2012 ▸; Stöcker & Gomis-Rüth, 2013 ▸; Bond, 2019 ▸) named after the archetypal digestive enzyme astacin from the European freshwater crayfish *Astacus astacus* L., which was first described in 1967 (Pfleiderer *et al.*, 1967 ▸; Stöcker *et al.*, 1988 ▸, 1992 ▸; Stöcker & Yiallouros, 2013 ▸). Astacins are characterized by a central ∼200-residue zinc-dependent catalytic domain (CD), which occurs in >12 000 sequences from >1000 species of identified and putative family members grouped into family PF01400 within the PFAM database (Mistry *et al.*, 2021 ▸). Sequences are found consistently with Darwinian vertical descent throughout metazoans and, sporadically, up to the root of holozoans. They are absent from plants and viruses (Semenova & Rudenskaia, 2008 ▸) and are found to be scattered across bacteria, which suggests that they are xenologues resulting from horizontal gene transfer from eukaryotes (Koonin *et al.*, 2001 ▸; Keeling & Palmer, 2008 ▸). The structural characteristics of astacin CDs further place the family within the metzincin clan of MPs (Bode *et al.*, 1993 ▸; Stöcker *et al.*, 1995 ▸; Gomis-Rüth, Trillo-Muyo *et al.*, 2012 ▸; Cerdà-Costa & Gomis-Rüth, 2014 ▸) and family M12A of the MEROPS database (Rawlings & Bateman, 2021 ▸).

Astacins share a basic domain architecture consisting of an N-terminal signal peptide for secretion, a pro-peptide (PP) of variable length (from 34 residues in astacin to 486 residues in *Drosophila melanogaster* tolkin; Finelli *et al.*, 1995 ▸; Gomis-Rüth, Trillo-Muyo *et al.*, 2012 ▸; Arolas *et al.*, 2018 ▸) for zymogenic latency and the CD (Gomis-Rüth, Trillo-Muyo *et al.*, 2012 ▸). This core may be C-terminally extended by disparate modules, among which are linkers (LNK), CUB domains (found in the complement component C1r/1s, the embryonic sea urchin Uegf and bone morphogenetic protein 1; Bork & Beckmann, 1993 ▸; PF00431) and MAM domains (common to meprins, A5 receptor protein and tyrosine phosphatase μ; Cismasiu *et al.*, 2004 ▸; PF00629). Two astacins, namely a short 240-residue protein (*astl* gene; UniProt accession B4F319) and a long 403-residue protein (*astl-mam* gene; UniProt B4F320), were identified in *L. polyphemus*, recombinantly expressed and biochemically characterized (Becker-Pauly *et al.*, 2009 ▸, 2011 ▸). The short form was predominantly found in the eyes and the brain, which suggests a function in the nervous system, while the long form was ubiquitous (Becker-Pauly *et al.*, 2009 ▸). The short paralogue has the basic domain architecture of the family, while the long paralogue further contains an LNK and an MAM domain. Both astacins share 46% sequence identity within the PP and the CD, and their trypsin-activated forms showed proteolytic activity in gelatin zymography and in solution against azocasein and the extracellular matrix proteins fibronectin, type IV collagen, gelatin and laminin, but not triple-helical collagen (Becker-Pauly *et al.*, 2009 ▸). Finally, consistent with the horseshoe crab being a chelicerate, these astacins were found to be closer to an orthologue from the brown spider *Loxoceles intermedia* in a phylogenetic analysis than to the crustacean orthologs from the crayfish *A. astacus* and the shrimp *Panaeus vannamei* (Becker-Pauly *et al.*, 2009 ▸).

Here, we crystallized the zymogen of the long paralogue, hereafter referred to as pLAST-MAM, and solved its crystal structure. Our results provide structural and molecular insight into the latency mechanism of the currently evolutionarily oldest holozoan astacin.

## 2. Methods

### 2.1. Protein crystallization

The pLAST-MAM zymogen was obtained by recombinant expression in *Trichoplusia ni* High Five insect cells, purified as described in Becker-Pauly *et al.* (2009 ▸) and subsequently concentrated in a Vivaspin device using a polyethersulfone membrane with 10 kDa cutoff (Vivaproducts). We screened for crystallization conditions using the sitting-drop vapour-diffusion method at the joint IBMB/IRB Automated Crystallo­graphy Platform (https://www.ibmb.csic.es/en/facilities/automated-crystallographic-platform). Reservoir solutions were prepared using a Tecan Freedom EVO robot and were dispensed into 96 × 2-well MRC plates (Innovadyne Technologies). A Phoenix/RE robot (Art Robbins) administered crystallization nanodrops consisting of 100 nl each of protein and reservoir solution. Crystallization plates were subsequently incubated at 4 or 20°C in Bruker steady-temperature crystal farms. Successful initial conditions were refined and scaled up to the microlitre range in 24-well Cryschem crystallization dishes (Hampton Research) whenever possible. Optimal crystals of the protein at ∼7 mg ml^−1^ in 50 m*M* HEPES pH 7.0 were obtained at 20°C using 0.1 *M* bicine pH 9.0, 10% polyethylene glycol (PEG) 40 000, 2% dioxane as the reservoir solution. Crystals were thin and fragile rectangular plates, which were harvested using cryo-loops (Molecular Dimensions), rapidly passed through a cryo-buffer consisting of reservoir solution plus 20%(*v*/*v*) glycerol and flash-vitrified in liquid nitrogen for transport and data collection.

### 2.2. Diffraction data collection and processing

X-ray diffraction data were collected on 18 April 2010 using an ADSC Quantum 315r detector on beamline ID29 of the ESRF synchrotron, Grenoble, France. Diffraction data were processed using *XDS* (Kabsch, 2010 ▸) and *XSCALE*, and were transformed to MTZ format using *XDSCONV* for use with the *Phenix* (Liebschner *et al.*, 2019 ▸) and *CCP*4 (Winn *et al.*, 2011 ▸) suites. Analysis with *phenix.xtriage* within *Phenix* revealed an absence of translational noncrystallographic symmetry (NCS) and no significant twinning according to the *L*-test. The crystals contained two monomers in the asymmetric unit and Table 1 ▸ provides essential statistics on data collection and processing.

### 2.3. Structure solution and refinement

The structure of pLAST-MAM was solved by molecular replacement using the *Phaser* crystallographic software (McCoy *et al.*, 2007 ▸) and a homology model for the CD and MAM domain predicted with *AlphaFold* (Jumper *et al.*, 2021 ▸). After several trials, we could only obtain correct solutions by searching with the domains separately, *i.e.* two for the CD but only one for the MAM domain. Those for the CD corresponded to Eulerian angles of α = 54.2, β = 54.0, γ = 116.3 and cell-fraction translation values of *x* = 0.106, *y* = 0.002, *z* = 0.210 for one protomer and α = 261.5, β = 125.5, γ = 297.0, *x* = 0.419, *y* = 0.884, *z* = 0.303 for the second protomer. The corresponding values for the MAM moiety were α = 64.8, β = 106.0, γ = 172.9, *x* = 0.285, *y* = 0.751, *z* = 0.991. These solutions had a final translation-function *Z*-score of 17.1 and a global log-likelihood gain after refinement of 782.

The suitably rotated and translated molecules were subjected to the *phenix.autobuild* protocol (Terwilliger *et al.*, 2008 ▸) within *Phenix*, which yielded a greatly improved Fourier map for manual model building with *Coot* (Casañal *et al.*, 2020 ▸). The latter alternated with crystallographic refinement using the *phenix.refine* protocol (van Zundert *et al.*, 2021 ▸) and *BUSTER* (Smart *et al.*, 2012 ▸), which both included translation/liberation/screw motion and NCS restraints, until completion of the model. The latter comprised residues Glu22–Cys403 of protomer *A* and Glu22–Gly246 of protomer *B*, each with a catalytic zinc ion plus one tentatively assigned magnesium cation, one diethylene glycol molecule, one triethylene glycol molecule, two glycerol molecules and 229 solvent molecules. The occupancy of LNK and MAM of protomer *A* refined to 87%. Table 1 ▸ provides essential statistics on the final refined model, which was validated through the wwPDB validation service (https://validate-rcsb-1.wwpdb.org/validservice). The coordinates can be retrieved from the Protein Data Bank (https://www.wwpdb.org/) as entry 8a28.

### 2.4. Miscellaneous

Structure superpositions were performed with *SSM* (Krissinel & Henrick, 2004 ▸) within *Coot*. Figures were prepared using *UCSF Chimera* (Goddard *et al.*, 2018 ▸). Protein interfaces and intermolecular interactions were analysed using *PDBePISA* (https://www.ebi.ac.uk/pdbe/pisa; Krissinel & Henrick, 2007 ▸) and verified by visual inspection. For this, the interacting surface of a complex was taken as half of the sum of the buried surface areas of either molecule.

## 3. Results and discussion

### 3.1. Overall crystal arrangement

**Figure 1:**
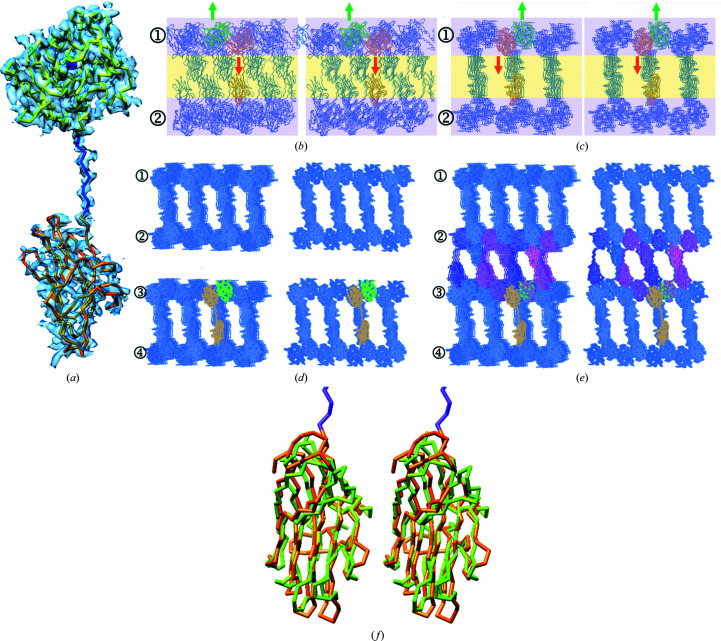
Crystal features. (*a*) Experimental structure of pLAST-MAM protomer *A* in C^α^ representation superposed with the final (2*mF*_obs_ − *DF*_calc_)-type Fourier map contoured at 0.5σ above the threshold. The CD is in yellow with the zinc ion as a magenta sphere, the LNK is in purple and the MAM domain is in orange. (*b*) Crystal packing viewed down the *x* axis of the crystal in cross-eyed stereo. The CDs of protomers *A* (orange C^α^ trace) and *B* (green C^α^ trace), together with their symmetry mates (purple C^α^ traces), are in planes (1 and 2; pink background) that parallel the *xy* plane of the crystal. The two CDs are in a relative upside-down conformation, so their respective C-termini point either downwards (protomer *A*, orange arrow) or upwards (protomer *B*, green arrow). The MAM domains of protomer *A* and its symmetry mates occupy the space between the CD sections (yellow background). (*c*) Same as (*b*) but viewed down the crystal *y* axis, *i.e.* after a vertical 90° rotation. Note that each LNK interacts in extended conformation with a symmetric MAM domain to build up the crystal in the section with the yellow background between the CD planes. Further contacts are observed between symmetric MAM domains. (*d*) Stereoview down the *y* axis as in (*c*) showing four CD sections (1–4) as they occur in the crystal. No atoms are found in the space between sections 2 and 3 as the LNK and MAM domain of the *B* protomers (CD in green C^α^ trace) is disordered in the final model. (*e*) Same as (*d*) after superposing protomers *A* and *B* using their respective CDs. The LNK and MAM domain of protomer *B* and its symmetry mates (in purplish colours) would establish the crystal contacts required to build up the crystal in the space between sections 2 and 3. Thus, these moieties must be present in the crystal but are disordered. (*f*) Superposition in stereo of the MAM domain of pLAST-MAM predicted with *AlphaFold* and refined against experimental diffraction data (LNK in purple, MAM domain in orange) and the experimental MAM domain of human meprin β (chartreuse; PDB entry 4gwm; Arolas *et al.*, 2012 ▸).

To prevent autolysis, pLAST-MAM was recombinantly expressed in insect cells as a point mutant in which the general base/acid glutamate for catalysis (Arolas *et al.*, 2018 ▸; E^140^; residues are given as single-letter codes with numbering in superscript according to UniProt B4F320; other proteins are numbered in subscript) was replaced by alanine to create a catalytically impaired variant. This strategy has often been employed in the past to prevent autolysis when crystallizing MP zymogens (see Table 1 in Arolas *et al.*, 2018 ▸). pLAST-MAM crystals with two protomers (*A* and *B*) in the crystallographic asymmetric unit were obtained in 2010 (Table 1 ▸) but the structure was only solved very recently using a homology model predicted by *AlphaFold* (Jumper *et al.*, 2021 ▸) for molecular replacement. After extensive calculations with the whole molecule and separate domains, the two CDs (N^49^–C^244^) could confidently be placed, rebuilt and refined, as well as the respective PPs (defined for E^22^–K^48^). In contrast, the LNK (F^245^–D^257^) and MAM (F^258^–C^403^) moieties were flexible and only those of protomer *A* could be placed in the structure. Moreover, crystallographic refinement revealed that the final Fourier map was discontinuous in several places in the MAM domain owing to this flexibility (Fig. 1 ▸ *a*). Indeed, while the CDs showed average thermal displacement parameters (*B* factors) of 60 and 73 Å^2^ for protomers *A* and *B*, respectively, the segment spanning LNK and MAM of protomer *A* had an average *B* factor of 116 Å^2^ after occupancy refinement to 87%.

Inspection of the crystal packing revealed that the two CDs form tight layers parallel to the *xy* plane of the crystal with their respective crystallographic symmetry mates (1 and 2 in Figs. 1 ▸ *b* and 1 ▸ *c*). They are in a relative upside-down conformation, so that the C-termini protrude either above or below the CD layer. In the case of the *A* protomers, LNK and MAM project into the space between CD sections and make interactions with symmetric MAM and LNK moieties from the CD layer beneath, respectively, which are required to form the crystal (Figs. 1 ▸ *b* and 1 ▸ *c*). In contrast, the space between CD sections into which the C-termini of the *B*-protomer CDs point (sections 2 and 3 in Fig. 1 ▸ *d*) does not contain any atoms and thus lacks crystal contacts owing to the missing LNKs and MAMs. However, when superposing the full-length protomer *A* on protomer *B* by their respective CDs, the LNK and MAM moieties adopt a very similar arrangement in the space between the two CD layers to that seen in the *A* protomers (sections 2 and 3 in Fig. 1 ▸ *e*). Thus, LNK and MAM of the *B* protomers must also be present in the crystal to establish the intermolecular contacts necessary to build the crystal. Overall, we conclude that while both LNK–MAM moieties are very flexible and adopt several slightly different orientations that are able to assemble the crystal, those of protomer *A* are somewhat more rigid, so they are grossly defined in the final Fourier maps. In contrast, those of protomer *B* are so flexible that the density is too poor to confidently place them.

Thus, given the poor definition of the MAM domains, we will concentrate the discussion hereafter on the PP and CD moieties of the zymogen (referred to here as pLAST) and the mature CD (LAST) of protomer *A*, and the mechanism of latency in the context of other structurally characterized astacin zymogens. Suffice to say that the predicted structure of the MAM domain of pLAST-MAM is very similar to that of the human astacin-family member meprin β except for some loops (Fig. 1 ▸ *f*). For a discussion of the architecture and features of these domains, please refer to Cismasiu *et al.* (2004 ▸), Aricescu *et al.* (2006 ▸, 2007 ▸), Arolas *et al.* (2012 ▸), Yelland & Djordjevic (2016 ▸) and Eckhard *et al.* (2021 ▸).

### 3.2. Structure of the zymogen

**Figure 2:**
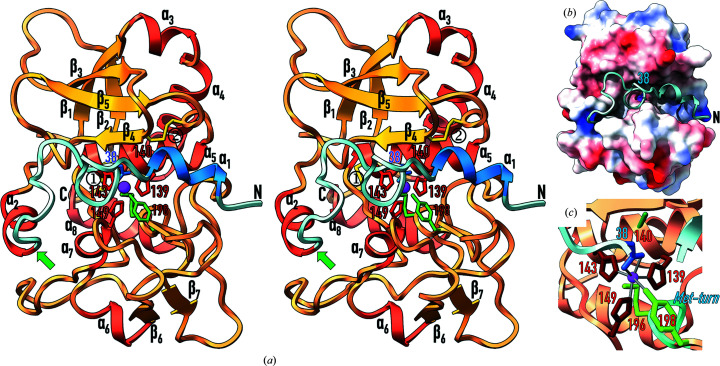
Structural features of pLAST. (*a*) Ribbon-type plot of the PP (blue ribbon) and the CD (orange/red ribbon) of the *Limulus* astacin zymogen in cross-eyed stereo. The regular secondary-structure elements (helices α1–α8 in blue/brick and β-strands β1–β7 in orange) are depicted and labelled, as are the N-terminus and the C-terminus. The activation cleavage site K^48^–N^49^ is pinpointed by a green arrow and the catalytic zinc is pictured as a purple sphere. Residues engaged in zinc binding are shown with their side chains as sticks and labelled (D^38^ in blue; H^139^, H^143^ and H^149^ in red; Y^198^ in green), as are the Met-turn methionine (M^196^ in green), the general base/acid glutamate (E^140^) mutated to alanine (in red) and the two disulfide bonds (yellow; 1, C^90^–C^244^; 2, C^112^–C^131^). (*b*) Structure of the zymogen showing the electrostatic surface of the CD and the PP as a pale blue ribbon traversing the deep and extended active-site cleft in the reverse direction to a substrate. The side chain of the ‘aspartate-switch’ residue D^38^ is shown and labelled. (*c*) Close-up view of (*a*) depicting the distorted octahedral coordination sphere of the catalytic zinc ion. The N^ɛ2^ atoms of H^139^ and H^149^ as well as the O^δ1^ and O^δ2^ atoms of D^38^ lie in a plane with the metal. The H^143^ N^ɛ2^ and, more distantly, Y^198^ O^η^ atoms occupy the apical positions. The Met-turn, which includes M^196^, is pictured as a cyan ribbon and labelled.

**Figure 3:**
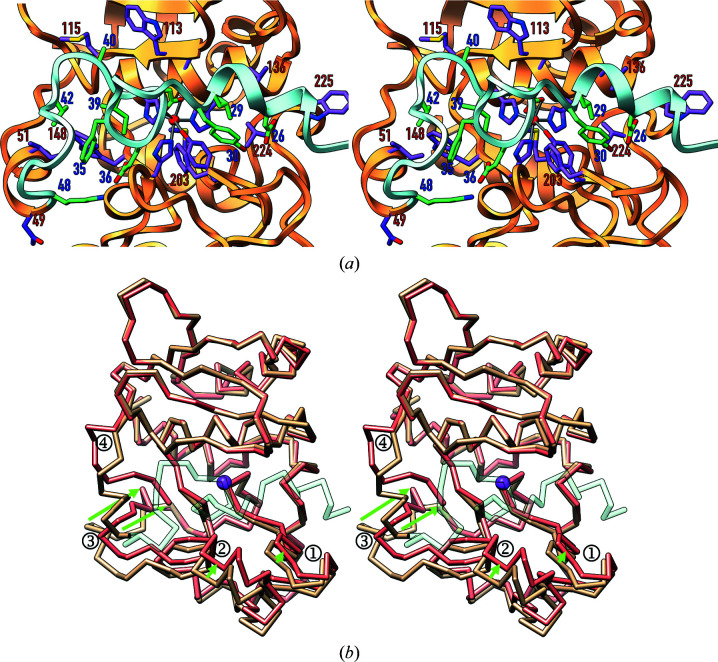
Active-site cleft details and proposed activation mechanism of pLAST. (*a*) Close-up view of Fig. 2 ▸(*a*) in stereo depicting residues engaged in the PP–CD interaction as sticks with C atoms in green (PP; blue labels) or plum (CD; red labels). The labels of the residues shown in Fig. 2 ▸(*c*) have not been included for clarity. (*b*) Superposition in stereo of the C^α^ traces of the experimental structure of pLAST (in tan for the CD moiety and semi-transparent aquamarine for the PP) and the *AlphaFold* homology model of LAST (in salmon) to illustrate the proposed activation mechanism. Small differences are found in segments G^199^–D^206^ (1) and E^150^–E^179^ (2) owing to a closing motion that slightly narrows the cleft. Large differences are encountered for the ‘activation segment’ (3; P^180^–N^187^) and the first seven residues of the mature CD (4; N^49^–L^56^). Green arrows pinpoint the proposed movements upon maturation.

The pLAST moiety subdivides into three segments when viewed in the standard orientation of MPs (Gomis-Rüth, Botelho *et al.*, 2012 ▸): the N-terminal PP (E^22^–K^48^), an upper N-terminal subdomain (NTS) of the CD (N^49^–G^146^) and a lower C-terminal subdomain (CTS) of the CD (F^147^–C^244^) (Fig. 2 ▸ *a*). The PP runs along the front surface of pLAST from right to left and features helix α1 on the primed side of the cleft (substrate and active-site subsite terminology based on Schechter & Berger, 1967 ▸; Gomis-Rüth, Botelho *et al.*, 2012 ▸). It adopts a wide loop structure protruding from the cleft between L^29^ and D^38^ (Fig. 3 ▸ *a*), which is stabilized by two intra-main-chain hydrogen bonds (H^31^ N–G^37^ O and F^35^ O–I^39^ N). The intervening residues are included in a ‘PP motif’ found in astacins (F-E/Q-G-D-I; Gomis-Rüth, Trillo-Muyo *et al.*, 2012 ▸), F^35^-E-G-D-I^39^ in pLAST (Becker-Pauly *et al.*, 2009 ▸). For I^39^–G^41^, the polypeptide adopts an extended conformation along the nonprimed side of the cleft before turning 90° downwards for V^42^–Y^45^ and then leftwards for Y^45^–D^47^. Thereafter, the peptide containing the primary activation cleavage site (K^48^–N^49^) enters into the CD, which adopts a helical conformation for K^48^–H^54^ (α2; Figs. 2 ▸ *a* and 3 ▸ *a*).

As in other astacins, the 195-residue CD divides into an NTS and a CTS of approximately equal size (Fig. 2 ▸ *a*). The NTS is rich in regular secondary structure and consists of a five-stranded arched and twisted β-sheet (β1–β5), the strands of which parallel the active-site cleft except for the lowermost (β4), which is antiparallel and frames the upper rim of the cleft. The concave face of the sheet accommodates three helices (α3–α5), among which are a ‘backing helix’ (α4) and an ‘active-site helix’ (α5) that are characteristic of astacins and metzincins in general (Bode *et al.*, 1993 ▸; Stöcker *et al.*, 1993 ▸; Stöcker & Bode, 1995 ▸; Gomis-Rüth, 2009 ▸; Gomis-Rüth, Trillo-Muyo *et al.*, 2012 ▸; Cerdà-Costa & Gomis-Rüth, 2014 ▸; Arolas *et al.*, 2018 ▸). The active-site helix encompasses the first two-thirds of a conserved zinc-binding motif (H^139^-E-*X*-*X*-H-*X*-*X*-G-*X*-*X*-H^149^ in pLAST) found in astacins and other metzincins, which features three metal-binding histidines and the general base/acid glutamate, here replaced with an alanine (see above and Fig. 2 ▸ *c*). At the glycine of the motif (G^146^), the polypeptide undergoes a sharp downwards turn to enter the CTS, which in contrast to the NTS is more irregular. It contains two short helices (α6 and α7) and the short β-ribbon β6β7 in addition to a ‘C-terminal helix’ (α8), which again is characteristic of metzincins. Of note is another conserved structural element of metzincins, the ‘Met-turn’, which is a tight 1,4-turn (S^194^–L^197^) encompassing the strictly conserved M^196^ (Fig. 2 ▸ *c*). Its side chain provides a hydrophobic pillow for the metal-binding site that is essential for the stability and function of metzincins (Tallant, García-Castellanos *et al.*, 2010 ▸). Immediately downstream of this methionine, Y^198^ provides the fourth zinc ligand of the CD through its somewhat more distant O^η^ atom. In other astacins, this residue is swung out upon substrate binding following a ‘tyrosine switch’ and its O^η^ atom participates in stabilization of the reaction intermediate during catalysis (Stöcker & Yiallouros, 2013 ▸). Finally, a disulfide bond links the back of the NTS with the C-terminal helix α8 of the CTS (C^90^–C^244^) and a second one links strand β4 with the loop connecting β5 and α5 (Lβ5α5) (C^112^–C^131^) (Fig. 2 ▸ *a*).

### 3.3. Mechanism of latency

Latency is achieved in pLAST by blocking access of substrates through the PP, which runs across the active-site cleft of the CD moiety in the opposite direction to a substrate (Figs. 2 ▸ *a*, 2 ▸ *b* and 3 ▸ *a*). This is a strategy to prevent untimely autolytic cleavage *in cis* (Khan & James, 1998 ▸; Arolas *et al.*, 2018 ▸). In addition, the polypeptide chain does not adopt an extended conformation as required for substrates to be cleaved (Tyndall *et al.*, 2005 ▸) but rather the aforementioned loop structure protrudes from the cleft (Fig. 2 ▸ *b*). This prevents a scissile bond from extending across cleft subsites S_1_ and 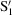 (Fig. 3 ▸ *a*), which is another mechanism to prevent undesired cleavage (Arolas *et al.*, 2018 ▸). The surface occluded by the PP–CD interaction spans 1207 Å^2^, which is in the range reported for protein–protein complexes (∼380–3390 Å^2^; Chen *et al.*, 2013 ▸), and has a solvation free-energy gain upon interface formation (Δ^i^ *G*) of −16.9 kcal mol^−1^ (Krissinel & Henrick, 2007 ▸), indicating a strong interaction. Participating structural elements include the entire PP and segments N^49^–V^52^, D^110^–V^116^, Y^129^–H^143^, W^148^–N^152^, S^170^–M^178^, Y^198^–T^208^ and P^223^–K^226^ of the CD, with the establishment of 20 electrostatic inter­actions and hydrophobic contacts between 17 pairs of residues of either moiety (Table 2 ▸).

The primary activation site of pLAST (K^48^–N^49^) is inserted within short helix α2 and buried in the zymogen, thus preventing access by activating enzymes in a similar fashion as found in pro-astacin (Guevara *et al.*, 2010 ▸). Moreover, K^48^ N^ζ^ makes strong interactions with Y^173^ O (2.7 Å apart) and N^176^ O (3.1 Å) of the CD and with E^36^ O^ɛ2 ^(2.7 Å) of the PP motif, which likewise hinder activation. The latter interaction is reminiscent of the double salt bridge between an arginine and an aspartate in a PP motif found in matrix metallopeptidase (MMP) zymogens (P-R-C-G-*X*-P-D; van Wart & Birkedal-Hansen, 1990 ▸; Springman *et al.*, 1990 ▸; Tallant, Marrero *et al.*, 2010 ▸; Arolas *et al.*, 2018 ▸). Moreover, the activation-scissile-bond N atom is bound to D^47^ O^δ2^ (2.8 Å) within the PP, so the activation site is additionally protected in the zymogen. All of these findings support the maturation of pLAST requiring partial unfolding of the segment flanking the activation site and/or preliminary cleavages, as described for crayfish astacin (Yiallouros *et al.*, 2002 ▸; Guevara *et al.*, 2010 ▸).

The most relevant element for latency is D^38^, which binds the catalytic zinc in a bidentate manner through its O^δ1^ (2.2 Å) and O^δ2^ (2.4 Å) atoms (Fig. 2 ▸ *c*), thus replacing the catalytic solvent required for catalysis in mature MPs (Arolas *et al.*, 2018 ▸). This aspartate is embedded in the PP motif and contributes to a distorted octahedral metal coordination sphere together with H^139^ N^ɛ2^ (2.1 Å) and H^149^ N^ɛ2^ (2.1 Å) in plane with the cation and with H^143^ N^ɛ2^ (2.1 Å) and Y^198^ O^η^ (3.3 Å) in the apical positions. Thus, D^38^ functions as an ‘aspartate switch’ for latency maintenance as described previously for crayfish astacin (Guevara *et al.*, 2010 ▸) and human meprin β (Arolas *et al.*, 2012 ▸) within the astacins (see below) and for fragilysin-3 (Goulas *et al.*, 2011 ▸) and the bacterial MMP karilysin (Cerdà-Costa *et al.*, 2011 ▸) within other metzincins (Arolas *et al.*, 2018 ▸).

### 3.4. Proposed mechanism of activation

The archetypal astacin from crayfish, which like the horseshoe crab is an arthropod, represents the evolutionarily closest orthologue of LAST with a known mature structure (Bode *et al.*, 1992 ▸). Indeed, 157 C^α^ atoms from these proteins superpose with a core root-mean-square deviation (r.m.s.d.) of 1.3 Å (38% sequence identity). Moreover, a predicted homology model of LAST was obtained with *AlphaFold* (Jumper *et al.*, 2021 ▸), which showed most of the common features in relevant segments described for mature astacin. It had an average predicted local distance difference test (pLDDT) value of >97, which is indicative of high reliability (Tunyasuvunakool *et al.*, 2021 ▸). Thus, this model is taken hereafter as a working model of mature *Limulus* astacin.

Superposition of the pLAST structure and the LAST model (Fig. 3 ▸ *b*) reveals that the CD moieties mostly coincide. In particular, the NTSs match best, with an r.m.s.d. of 0.93 Å for all 746 atoms of segment L^57^–H^149^. The metal-binding site and most of the active-site cleft would largely be preformed in the zymogen, as observed for other MP zymogens (Arolas *et al.*, 2018 ▸). Within the CTS, good agreement is observed for the segment E^188^–G^199^, which includes the Met-turn, and the entire C-terminal stretch from G^207^ to C^244^. Loop G^199^–D^206^, which frames the lower rim of the cleft, slightly deviates, with a maximal displacement of ∼2 Å that closes the cleft on the primed side upon activation. On the bottom of the nonprimed side of the cleft, E^150^–E^179^ would additionally undergo a closing motion of maximally ∼3 Å facilitated by a ∼10° rotation around W^198^. The largest deviation, however, is observed for the segment P^180^–N^187^, which conforms to a flexible ‘activation domain’ and would become significantly rearranged (Fig. 3 ▸ *b*), as described for other astacins (Guevara *et al.*, 2010 ▸) and the otherwise unrelated trypsin-like serine endopeptidases (Huber & Bode, 1978 ▸). This rearrangement would result from the displacement of N^49^–L^56^, which upon maturation cleavage at K^48^–N^49^ would become rotated outwards around the C^α^—C bond of L^56^. In this way, the seven preceding residues would be amply repositioned by up to ∼11 Å and penetrate the mature enzyme moiety, so the first three residues (N^49^-A^50^-I^51^) would be completely inaccessible to solvent, as reported for meprin β (see Section 3.5). Next, N^49^ would bind the ‘family-specific residue’ immediately after the third zinc-binding histidine (E^150^; Bode *et al.*, 1993 ▸; Gomis-Rüth, 2003 ▸), which in turn is held in place by internal salt bridges with R^237^ and R^153^ in the zymogen. This interaction could occur directly through the N^49^ N^δ2^ atom, as observed in meprin β (Arolas *et al.*, 2012 ▸). An alternative interaction through the α-amino group (N^49^ N) mediated by a solvent molecule, as observed in crayfish astacin (Bode *et al.*, 1992 ▸), is also conceivable. Moreover, the N^49^ O^δ1^ atom might also bind the R^237^ side chain. Overall, this scenario of a deeply buried mature N-terminus is very similar to that found in other astacins, in which the maturation mechanism has been structurally verified (see Section 3.5). This, in turn, provides confidence in the reliability of the LAST homology model.

### 3.5. Comparison with other astacin latency mechanisms

**Figure 4:**
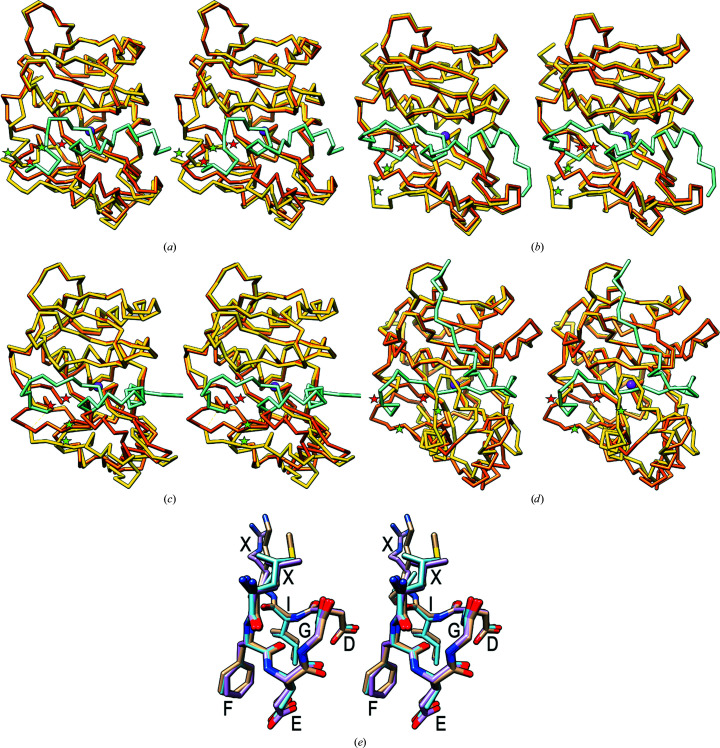
Activation of astacins with reported zymogen structures and a conserved PP motif. (*a*)–(*d*) Superposition in cross-eyed stereo of the C^α^ traces in standard orientation of the latent and mature forms of (*a*) *Limulus* astacin (latent, PDB entry 8a28; mature, *AlphaFold* model), (*b*) crayfish astacin [latent, PDB entry 3lq0 (Guevara *et al.*, 2010 ▸); mature, PDB entry 1ast (Bode *et al.*, 1992 ▸; Gomis-Rüth *et al.*, 1993 ▸)], (*c*) human meprin β [latent, PDB entry 4gwm (Arolas *et al.*, 2012 ▸); mature, PDB entry 4gwn (Arolas *et al.*, 2012 ▸)] and (*d*) *Myroides* sp. CSLBB myroilysin [latent, PDB entry 5gwd (Xu *et al.*, 2017 ▸); mature, PDB entry 5zjk (Ran *et al.*, 2020 ▸)]. The mature forms are in orange and the zymogens are in cyan (PP) and yellow (CD). The catalytic zinc ions are depicted as purple spheres. The PP of meprin β is N-terminally extended and runs across the front surface of a vicinal TRAF domain (not shown; Arolas *et al.*, 2012 ▸). The most relevant rearranged segments during maturation cleavage, the ‘activation segment’ and the mature N-terminal segment, are pinpointed by green and red stars in each structure, respectively. (*e*) Superposition of the segments encompassing the PP motif of astacins (F-E-G-D-I) in pLAST (C atoms in cyan), crayfish pro-astacin (C atoms in tan) and human pro-meprin β (C atoms in plum). Myroilysin lacks this motif.

To date, the crystal structures of crayfish pro-astacin (PDB entry 3lq0; Guevara *et al.*, 2010 ▸), human pro-meprin β (PDB entry 4gwm; Arolas *et al.*, 2012 ▸) and pro-myroilysin from two closely related bacterial species, *Myroides profundi* (PDB entry 5czw; Xu *et al.*, 2017 ▸) and *Myroides* sp. CSLB8 (PDB entry 5gwd; Xu *et al.*, 2017 ▸), have been reported, as well as their respective mature forms astacin (PDB entry 1ast; Bode *et al.*, 1992 ▸; Gomis-Rüth *et al.*, 1993 ▸), meprin β (PDB entry 4gwn; Arolas *et al.*, 2012 ▸) and myroilysin from *Myroides* sp. CSLB8 (PDB entry 5zjk; Ran *et al.*, 2020 ▸). The two proteins from *Myroides* are 99.6% identical, so only that from *Myroides* sp. CSLB8 will be discussed here. Of all these structures, only pro-meprin β spans additional domains downstream of the CD, namely an MAM and a TRAF domain (Arolas *et al.*, 2012 ▸). Pictures of the three zymogens superposed onto the mature forms, together with those of the pLAST structure and the LAST model, are provided in Figs. 4 ▸(*a*)–4 ▸(*d*).

In all cases, the mature N-terminus is buried inside the catalytic moiety and is bound to the family-specific glutamate of astacins either directly through an N-terminal asparagine (LAST and meprin β) or glycine (myroilysin) or mediated by a solvent molecule because the N-terminal segment is one residue shorter (astacin). The position of the new N-terminus in the zymogen and the mature moiety is very close in astacin (∼2 Å; Fig. 4 ▸ *b*), quite close in meprin β (∼6 Å; Fig. 4 ▸ *c*), farther apart in LAST (∼11 Å; Fig. 4 ▸ *a*) and farthest in myroilysin (∼17 Å; Fig. 4 ▸ *d*).

Detailed analysis of the four zymogen–mature enzyme pairs reveals that in all cases the PP is poor in regular secondary structure and adopts a mostly extended conformation that traverses the active-site cleft in the opposite direction to a substrate. In pro-myroilysin it is additionally elongated at the N-terminus and further extends along the front surface of the NTS (Fig. 4 ▸ *d*), while in pro-meprin β (Fig. 4 ▸ *c*) it runs in an extended conformation along a neighbouring TRAF domain on the right of the CD (not shown). In all cases, CTS regions framing the bottom of the active-site cleft on its nonprimed side constitute activation segments that undergo rearrangement upon maturation cleavage and repositioning of the new N-terminus. In astacin, only this activation segment (I_130_–E_139_, mature enzyme numbering according to PDB entry 1ast; add 49 for full-gene numbering; see UniProt P07584) is reorganized, while the rest of the molecule is preformed in the zymogen (Guevara *et al.*, 2010 ▸; Fig. 4 ▸ *b*). Next, LAST is most likely to undergo slight rearrangement of two segments (G^199^–D^206^ and E^150^–E^179^) in addition to the major movement of the activation segment (P^180^–N^187^; see Section 3.4 and Fig. 4 ▸ *a*). Meprin β, in turn, repositions most of its CTS (Q_164_–Y_211_ and L_199_–D_233_ according to UniProt Q16820; segment D_194_–L_199_ is disordered in the zymogen structure) in a concerted hinge motion that entirely closes the cleft at its bottom in response to maturation (Fig. 4 ▸ *c*). Finally, the largest deviation is observed in myroilysin, which rearranges its entire CTS except for the Met-turn and the C-terminal helix (Fig. 4 ▸ *d*). The segments affected are Q_155_–A_201_ and Y_210_–N_225_ (myroilysin numbering according to PDB entry 5gwd; see also UniProt A0A0P0DZ84). A large flap (N_160_–S_193_), which encompasses two helices, is folded back on top of the active-site cleft and traps the PP in the zymogen. Upon maturation, this flap is rotated to the right with a maximal displacement of ∼17 Å (measured at P_176_), thus liberating access to the cleft (Fig. 4 ▸ *d*).

Differences are also found in the residues blocking the zinc ion in the zymogen. The three metazoan proteins contain an aspartate within the PP motif, which is structurally conserved (Fig. 4 ▸ *e*), acting as an aspartate switch. In contrast, the bacterial enzyme lacks the PP motif and instead features a cysteine, which blocks the zinc according to a ‘cysteine-switch’ mechanism (Ran *et al.*, 2020 ▸; Xu *et al.*, 2017 ▸). Moreover, the polypeptide chain flanking the cysteine is in a canonical, extended conformation and does not adopt the loop of the PP motif. Overall, this is inversely reminiscent of MMPs, in which canonical vertebrate orthologues regulate latency according to a cysteine-switch mechanism (Springman *et al.*, 1990 ▸; van Wart & Birkedal-Hansen, 1990 ▸; Rosenblum *et al.*, 2007 ▸; Tallant, Marrero *et al.*, 2010 ▸; Arolas *et al.*, 2018 ▸), while the bacterial orthologue karilysin from the periodontopathogen *Tannerella forsythia* instead operates according to an asparate switch. As in astacins, MMPs are only found dispersedly outside animals, and it has been proposed that karilysin is a xenologue co-opted from a mammalian host through horizontal gene transfer facilitated by intimate interaction between the host and the colonizing bacterium (Cerdà-Costa *et al.*, 2011 ▸). A similar origin is conceivable for myroilysin within astacins given that *Myroides* spp. have been reported in several human body fluids and can trigger infection leading to soft-tissue infections in humans (Maraki *et al.*, 2012 ▸) and bacteraemia in a diabetic patient (Endicott-Yazdani *et al.*, 2015 ▸). Thus, as in MMPs, the latency mechanisms of holozoan orthologues and bacterial xenologues would also diverge in astacins.

## 4. Data availability

All data and reagents are freely available from the authors upon reasonable request.

## Supplementary Material

PDB reference: *Limulus polyphemus* astacin zymogen, 8a28

## Figures and Tables

**Table 1:** Crystallographic data Abbreviations: AU, crystallographic asymmetric unit; GOL, glycerol; PEG, diethylene glycol; PGE, triethylene glycol; RSRZ, real-space *R*-value *Z*-score. Values in parentheses are for the outermost resolution shell.

Beamline	ID29, ESRF
Space group	*C*2
Protomers per AU	2
*a*, *b*, *c* (Å)	115.83, 47.57, 236.40
α, β, γ (°)	90, 102.91, 90
Wavelength (Å)	0.97244
No. of measurements	316453
No. of unique reflections	49732
Resolution range (Å)	76.8–2.40 (2.54–2.40)
Completeness (%)	99.6 (98.6)
*R*_merge_ †	0.151 (1.431)
*R*_meas_ †	0.164 (1.564)
CC_1/2_ †	0.995 (0.594)
Average intensity‡	9.8 (1.9)
Wilson *B* factor (Å^2^)	53.7
Average multiplicity	6.4 (6.1)
Resolution range used for refinement (Å)	46.7–2.40
Reflections used (total/test set)	48988 (741)
Crystallographic *R*/*R*_free_ †	0.253/0.289
Contents of AU	
Non-H protein atoms	4801
Ionic ligands	2 Zn^2+^, 1 Mg^2+^
Waters	229
Non-ionic ligands	1 PGE, 1 PEG, 2 GOL
R.m.s.d. from target values	
Bond lengths (Å)	0.014
Angles (°)	1.26
Average *B* factor (Å^2^)	77.6
Analysis of protein contacts and geometry§	
Ramachandran favoured/outliers/all analyzed	550 [91%]/10/603
Bond-length/bond-angle/chirality/planarity outliers	0/1/0/0
Side-chain outliers	29 [5.8%]
All-atom clashes	43
Clashscore	4.5
RSRZ outliers§	131 [21.6%]
*F*_o_–*F*_c_ correlation	0.89 (0.88)
PDB code	8a28

† For definitions, see Einspahr & Weiss (2012 ▸).

‡ Average intensity is the 〈*I*/σ(*I*)〉 of unique reflections after merging according to *XSCALE* (Kabsch, 2010 ▸).

§ According to the wwPDB Validation Service (https://wwpdb-validation.wwpdb.org/validservice).

**Table 2:** Interactions between the pro-peptide (PP) and the catalytic domain (CD) of pLAST protomer *A*

PP	CD	Distance (Å)
Hydrogen bonds (<3.5 Å)		
E^22^ O	W^225^ N^δ1^	2.8
N^23^ N^δ2^	T^201^ O^γ1^	3.0
N^23^ N^δ2^	G^207^ O	2.9
D^26^ O^δ1^	W^225^ N	3.1
D^26^ O^δ2^	V^224^ N	3.0
P^28^ O	C^112^ N	2.9
E^36^ O	Y^198^ O^η^	2.7
E^36^ O^ɛ1^	H^149^ N^δ1^	3.1
E^36^ O^ɛ2^	N^152^ N^δ2^	2.9
D^38^ O	S^114^ N	3.0
D^38^ O^δ1^	Y^198^ O^η^	3.0
A^40^ N	S^114^ O	3.2
A^46^ O	Y^173^ O^η^	2.8
D^47^ O	I^51^ N	3.2
D^47^ O^δ1^	N^49^ N	2.8
D^47^ O^δ1^	A^50^ N	3.3
K^48^ N^ζ^	Y^173^ O	2.7
K^48^ N^ζ^	N^176^ O	3.1
Metallorganic bonds		
D^38^ O^δ1^	Zn999	2.2
D^38^ O^δ2^	Zn999	2.4
Van der Waals interactions (<4 Å)		
P^28^	C^112^	
P^28^	C^131^	
L^29^	L^136^	
L^29^	Y^198^	
L^29^	V^224^	
Y^30^	Y^198^	
Y^30^	A^202^	
Y^30^	F^203^	
L^34^	W^113^	
F^35^	I^51^	
F^35^	W^148^	
I^39^	W^148^	
I^39^	V^116^	
A^40^	W^113^	
A^40^	M^115^	
V^42^	I^51^	
Y^45^	Y^173^	
